# Prediction of chemo-response in serous ovarian cancer

**DOI:** 10.1186/s12943-016-0548-9

**Published:** 2016-10-19

**Authors:** Jesus Gonzalez Bosquet, Andreea M. Newtson, Rebecca K. Chung, Kristina W. Thiel, Timothy Ginader, Michael J. Goodheart, Kimberly K. Leslie, Brian J. Smith

**Affiliations:** 1 Department of Obstetrics and Gynecology, University of Iowa Hospitals and Clinics, University of Iowa, 200 Hawkins Dr, Iowa City, IA 52242 USA; 2 Holden Comprehensive Cancer Center, University of Iowa, Iowa City, IA USA; 3 Biostatistics, Holden Comprehensive Cancer Center, University of Iowa, Iowa City, IA USA

**Keywords:** Ovarian cancer, Chemo-response, Prediction model, Data integration, Individualized treatment

## Abstract

**Background:**

Nearly one-third of serous ovarian cancer (OVCA) patients will not respond to initial treatment with surgery and chemotherapy and die within one year of diagnosis. If patients who are unlikely to respond to current standard therapy can be identified up front, enhanced tumor analyses and treatment regimens could potentially be offered. Using the Cancer Genome Atlas (TCGA) serous OVCA database, we previously identified a robust molecular signature of 422-genes associated with chemo-response. Our objective was to test whether this signature is an accurate and sensitive predictor of chemo-response in serous OVCA.

**Methods:**

We first constructed prediction models to predict chemo-response using our previously described 422-gene signature that was associated with response to treatment in serous OVCA. Performance of all prediction models were measured with area under the curves (AUCs, a measure of the model’s accuracy) and their respective confidence intervals (CIs). To optimize the prediction process, we determined which elements of the signature most contributed to chemo-response prediction. All prediction models were replicated and validated using six publicly available independent gene expression datasets.

**Results:**

The 422-gene signature prediction models predicted chemo-response with AUCs of ~70 %. Optimization of prediction models identified the 34 most important genes in chemo-response prediction. These 34-gene models had improved performance, with AUCs approaching 80 %. Both 422-gene and 34-gene prediction models were replicated and validated in six independent datasets.

**Conclusions:**

These prediction models serve as the foundation for the future development and implementation of a diagnostic tool to predict response to chemotherapy for serous OVCA patients.

**Electronic supplementary material:**

The online version of this article (doi:10.1186/s12943-016-0548-9) contains supplementary material, which is available to authorized users.

## Background

Epithelial ovarian cancer (OVCA) has the highest mortality rate of all gynecologic cancers [1]. The most common histological subtype of OVCA is serous [2]. The majority of patients present with advanced disease at diagnosis and, while some benefit from a treatment combining cytoreductive surgery and chemotherapy [3], nearly a third of patients with serous OVCA will not respond to this initial treatment and die from disease within one year after diagnosis [1, 4]. Despite significant research directed at understanding the biology of OVCA [5, 6], outcomes remain poor for a majority of patients, particularly those who do not respond to initial chemotherapy. A major limitation is the lack of validated biomarkers that can effectively predict response to chemotherapy [7, 8].

Previous attempts to define predictors of response to treatment have been limited by number of patients included, mixture of histological types and stages, and lack of validation in independent sets [9, 10]. In contrast, breast cancer gene signatures have been identified that can accurately predict recurrence [11] and chemotherapeutic response [12, 13]. These signatures were subsequently validated in independent clinical studies [13–15]. For example, one of these signatures, OncotypeDx, used 600 cases to create an association model and validated it in an additional 400 cases [11, 12]. Currently, there is no similar clinically available test for OVCA to identify which patients will respond to initial treatment [16].

In recently published studies using the Cancer Genome Atlas (TCGA) serous OVCA database [17], we identified a robust molecular signature associated with chemo-response by integrating publicly available biological and clinical data from 450 serous OVCA patients. This yielded a 422-gene molecular signature that was replicated in five independent gene expression experiments [18]. The contributing data used to identify this signature included gene expression, gene copy number alteration, gene mutations, DNA methylation, and miRNA profiles, all of which are available in TCGA dataset for serous OVCA. The presence of a strong association between the 422-gene signature and chemo-response from our previous work, though, does not imply that the signature also is predictive of chemo-response [9].

Therefore, the main objective of the present study was to determine the performance of the 422-gene signature as a predictor of chemo-response in serous OVCA. We also optimized and determined which of the elements of the signature contributed more to all prediction models. In this process, we identified a smaller set of 34 genes (the “optimized” set) from the original 422 signature that are predictive of response and that replicated the area under the curve (AUC) of the original complete gene set. Our data demonstrate that both the complete and the optimized models are predictive of outcome and are now replicated and validated in independent datasets.

## Methods

### Patients and data collection for prediction model

All data collection and processing, including the consenting process, were performed after approval by all local institutional review boards and in accord with the TCGA Human Subjects Protection and Data Access Policies, adopted by the National Cancer Institute (NCI) and the National Human Genome Research Institute (NHGRI).

**Fig. 1:**
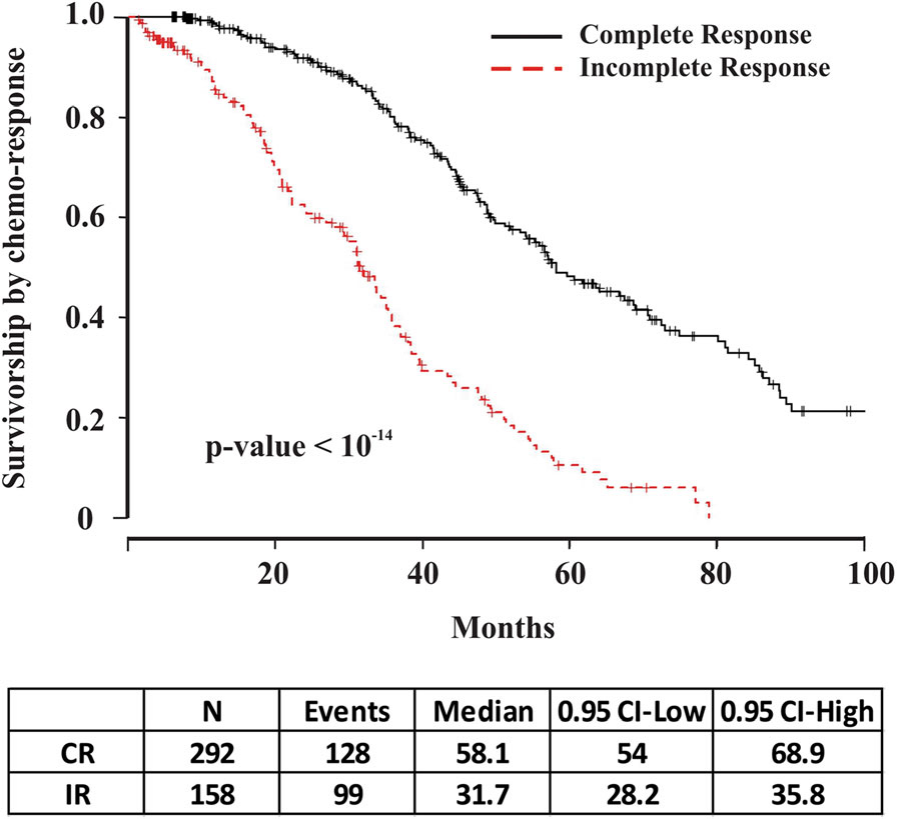
Survivorship by chemo-response in serous OVCA TCGA data. Chemo-response was the most significant factor in the multivariable analysis for survival. Complete responders (CR) have a median survival 2 years greater than IR

Patients with serous OVCA in TCGA were utilized to create a prediction model in the testing dataset, and were divided into two categories: complete responders (CR) and incomplete responders (IR). Clinical complete response (CR) was defined as progression-free survival 6 months after the first platinum-based treatment. In patients with incomplete response (IR), the disease either not did respond or progressed during treatment (refractory), or recurred within 6 months of treatment completion (resistant) [4, 19]. Patients defined as IR in our study are also clinically referred to as ‘platinum-resistant’ [20], with direct implications for treatment and prognosis. In the TCGA dataset, there were 292 patients classified as CR and 158 classified as IR. Table 1 describes the clinical characteristics of these patients. Chemo-response was the most significant prognostic factor for survival in multivariable analysis by Cox proportional hazards regression (*p*-value < 10^−14^), and patients with IR had a significantly decreased median survival compared to CR patients (Fig. 1) [18].

**Table 1:** Clinical data from TCGA patients

	CR	IR	*p*-value*
Number of Patients	292	158	
Age (Avg.)	60	59.6	N.S.
Grade			N.S.
Grade 1	4	1	
Grade 2	35	18	
Grade 3	246	135	
Stage			*p* < 0.01
Stage I	10	3	
Stage II	19	1	
Stage III	224	123	
Stage IV	39	29	
Surgical outcome			N.S.
Optimal (<1 cm residual)	207	92	
Suboptimal (>1 cm residual)	52	57	
Optimal Treatment			*p* < 0.001
Optimal (Surgery + 6 cycles)	179	66	
Suboptimal	113	92	

*Multivariable analysis of TCGA clinical variables: Only FIGO stage and optimal treatment (including optimal surgery AND 6 cycles of platinum-based chemotherapy) were independently associated with chemo-response in serous OVCA

### Gene signature and prediction analysis

We previously identified a 422-gene signature that is robustly associated with chemo-response [18]. To assess predictive performance of this signature, we applied the ‘*Classification for MicroArrays*’ (CMA) to TCGA serous OVCA data. CMA is a statistical tool designed to construct and evaluate classifiers (or prediction models) derived from microarray experiments using a large number of standard methods [21] and the R environment for statistical computing (www.r-project.org) [22].

Of the different methods available in the CMA package [21] to perform the analysis, nine methods consistently handle missing values, lower number of samples, and compute AUCs without reporting any errors: random forest [23], least absolute shrinkage and selection operator (Lasso) [24], Elastic Net [24], prediction analysis for microarrays (PAM) [25], diagonal discriminant analysis [26], partial least squares (PLS) [27], PLS - random forest [27], penalized logistic regression [28], and PLS - logistic regression [27]. We used these nine methods for the rest of the study to compare the predictive performance of all of the different datasets and for both the complete and optimized models. Two other available methods, linear and quadratic schrinkage, could not compute AUC. Fisher’s discriminant analysis could not handle more variables than subjects; neural networks was unstable/difficult to tune and interpret; k-nearest neighbors and support vector machines could not tune and evaluate AUCs.

Initially, all 422 genes associated with chemo-response in serous ovarian cancer [18] were utilized to construct prediction models, termed 422-gene prediction models. To assess how accurately the groups (CR and IR) were predicted, and to avoid over-fitting, cross-validation was used (internal validation of the classifier) [29]. The predictive performance was computed with corrections for TCGA batch-effect and to account for two other variables independently associated with chemo-response in serous OVCA (FIGO stage classification and optimal treatment, Table 1) [10]. Sensitivity, specificity and AUC of the predictor/classifier were also calculated. For each of the AUC measurements, we also computed a 95 % confidence interval (CI) to compare different models and different methods of classification. To illustrate the performance of the predictor in classifying chemo-response, a receiver operating characteristic (ROC) curve was generated. These analyses also facilitated comparison of the performance of the predictor models across independent serous OVCA datasets and assessed how consistently the models predicted chemo-response in OVCA patients based on sensitivity, specificity, misclassification rate, and AUC. Finally, we identified which patients were more likely to be misclassified and the clinical characteristics that were associated with misclassification.

### Selection of most informative genes of prediction models

We focused on the selection of informative genes, because the composition of prediction models is paramount for their performance [9]. The selection process was performed with all available methods in the software package: two-sample *t*-test; Welch modification of the *t*-test; Wilcoxon rank sum test; F-test; Kruskal-Wallis test; “moderated” t and F test, respectively, using the package ‘limma’ in R statistics; one-step Recursive Feature Elimination (RFE) in combination with the linear support vector machines (SVM); random forest variable importance measure; least absolute shrinkage and selection operator (or Lasso); the regularized regression method or elastic net; component-wise boosting; and ad-hoc “Golub” criterion [21]. Using the gene selection tool, each gene was ranked depending on its relative importance in prediction models. These genes were ordered based on their rank and their relative ‘weight’ in the prediction process, and the prediction model analysis was applied by including only those genes that had been ranked at least once (one ‘hit’) by each method. These models, containing only the 34 selected and more informative genes, were termed 34-gene prediction models and comprised the optimized gene set as compared to the complete gene set.

### Data retrieval for replication and validation analyses

Validation and replication of the prediction models was performed using datasets in the Gene Expression Omnibus (GEO) and the European Bioinformatics Institute, part of the European Molecular Biology Laboratory (EMBL-EBI), that contain gene expression paired with treatment response data (Table 2). Databases were downloaded in their raw state to maximize platform and annotation information, and then data were normalized. Response to therapy variables were coded to make outcomes comparable with TCGA: CR and IR. Also, patients that underwent optimal debulking (with largest residual disease of <1 cm) and completed six cycles of platinum-based therapy were considered to have ‘optimal treatment’. Lesser treatments were considered suboptimal. This analysis was performed because optimal treatment and FIGO stage of disease were also significantly and independently associated with chemo-response in TCGA (Table 1). Both clinical variables were collected, when available, and assessed for association to chemo-response in these new datasets in order to account for them in the prediction analysis. Also, batch-effect, if available, was accounted for to correct for any bias, as was also performed in the initial prediction model using the TCGA dataset.

**Table 2:** Publicly available GEO datasets of patients with serous OVCA used for validation/replication of prediction models

Repositories	Number of patients		Study Names	References
	CR	IR		
GEO accession number				
GSE23554 GSE3149	90	37	MCC	Marchion, 2011 [32] Bild, 2006 [33]
GSE9891	185	55	Australia	Tothill, 2008 [34]
GSE28739	20	30	Trinh	Trinh, 2011 [36]
GSE17260	93	17	Yoshihara	Yoshihara, 2010 [37]
GSE30161	32	23	Ferriss	Ferriss, 2012 [35]
EMBL-EBI accession number				
E-MTAB-386	64	41	Bentink	Bentink, 2012 [38]

### Replication and validation analyses

Initially, we replicated the prediction analysis of all 422 genes associated with chemo-response, or the complete prediction model, in all independent databases to assess how accurately chemo-response was predicted. Cross-validation was used for internal validation of the classifier, and predictive performance was computed with the same methods described for the TCGA dataset. Sensitivity, specificity, and AUC were calculated. Databases that contained information on all variables were later used for validation of the predictor/classifier of both the 422-gene and the 34-gene prediction models.

Validation of the 422-gene prediction models from TCGA data (the training set) and independent datasets (testing sets) was performed using the ‘prediction’ tool of the CMA package. Only independent datasets with information on all variables were used as testing sets. Sensitivity, specificity and AUC were used to measure the performance of the prediction model. Validation of the optimized 34-gene prediction models, including only the 34 most informative genes, was also performed in those independent datasets with information on all variables, and the same measures were used to assess the performance of the classifier.

### Software

Apart from the ‘*Classification for MicroArrays*’ (CMA) utilized for the prediction analysis and already described, other analyses (i.e., logistic regression, Cox regression, Kaplan-Meier survival estimation), were performed using R software for statistical computing and graphics and utilizing Bioconductor packages as open source software for bioinformatics (bioconductor.org). Differential gene expression analysis was performed using Biometric Research Branch (BRB) ArrayTools, an integrated package for visualization and statistical analysis that utilizes Excel (Microsoft, Redmond, WA) as a front end, and with tools developed in the R statistical system. BRB-ArrayTools were developed by Dr. Richard Simon and the BRB-ArrayTools development team. Associations of the 34 most informative genes with survival were estimated with a multivariate Cox regression model and reported as hazard-ratios along with 95 % confidence intervals, and likelihood ratio test was used to compare models of survival, also within R environment.

To identify biological processes and pathways over-represented in the selected group of genes, we performed pathway enrichment analyses with MetaCore 6.0 [30] (GeneGo Inc., MI), an integrated knowledge-based platform for pathway analysis of OMICs data and gene lists, and other R-based tools, such as clusterProfiler [31], which mines the KEGG database (Kyoto Encyclopedia of Genes and Genomes, www.genome.jp/kegg).

## Results

### *422-gene prediction model* with all signature genes

**Fig. 2:**
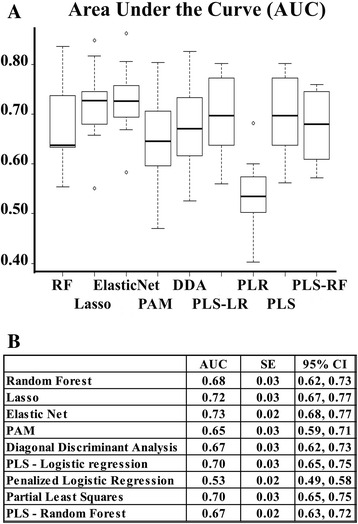
Area under the ROC curve (AUC) for the 422-gene prediction models. **a** Box plot representations of the AUC for the complete model by different methods. RF: Random forest; Lasso (least absolute shrinkage and selection operator); Elastic Net; PAM: Prediction analysis for microarrays; DDA: Diagonal discriminant analysis; PLS-LR: Partial least squares - Logistic regression; PLR: Penalized logistic regression; PLS: Partial least squares; PLS-RF: Partial least squares - Random forest. **b** Prediction performance measured in AUC, with their respective standard error, and confidence intervals (CI) by different methods using all 422 genes. PAM: Prediction analysis for microarrays; PLS: Partial least squares; Lasso: least absolute shrinkage and selection operator

Initially, we performed the prediction analysis of chemo-response including all 422 genes of the signature, which we refer to herein as the 422-gene prediction models. Figure 2 summarizes the predictive performance of all 422-gene prediction models. Their performance resulted in AUCs ranging from 53 to 73 %. Predictive performances of the 422-gene prediction models were not explained by prediction models constructed only with FIGO stage classification and optimal treatment, the clinical variables that have been independently associated with chemo-response (mean AUC around 58 %, Table 3).

**Table 3:**
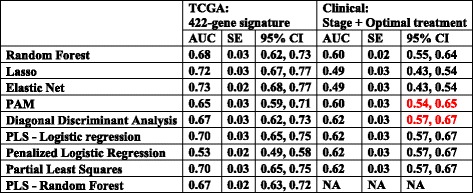
AUCs and their CI comparing the 422-gene prediction model and clinical prediction models

PAM: Prediction analysis for microarrays; PLS: Partial least squares; Lasso: least absolute shrinkage and selection operator; NA: not available (not computable)

CI of clinical prediction model WITH significant overlap with TCGA *422-gene prediction model* CI: in red

### Selection of the most informative genes and predictive performance of the *34-gene prediction model*

**Fig. 3:**
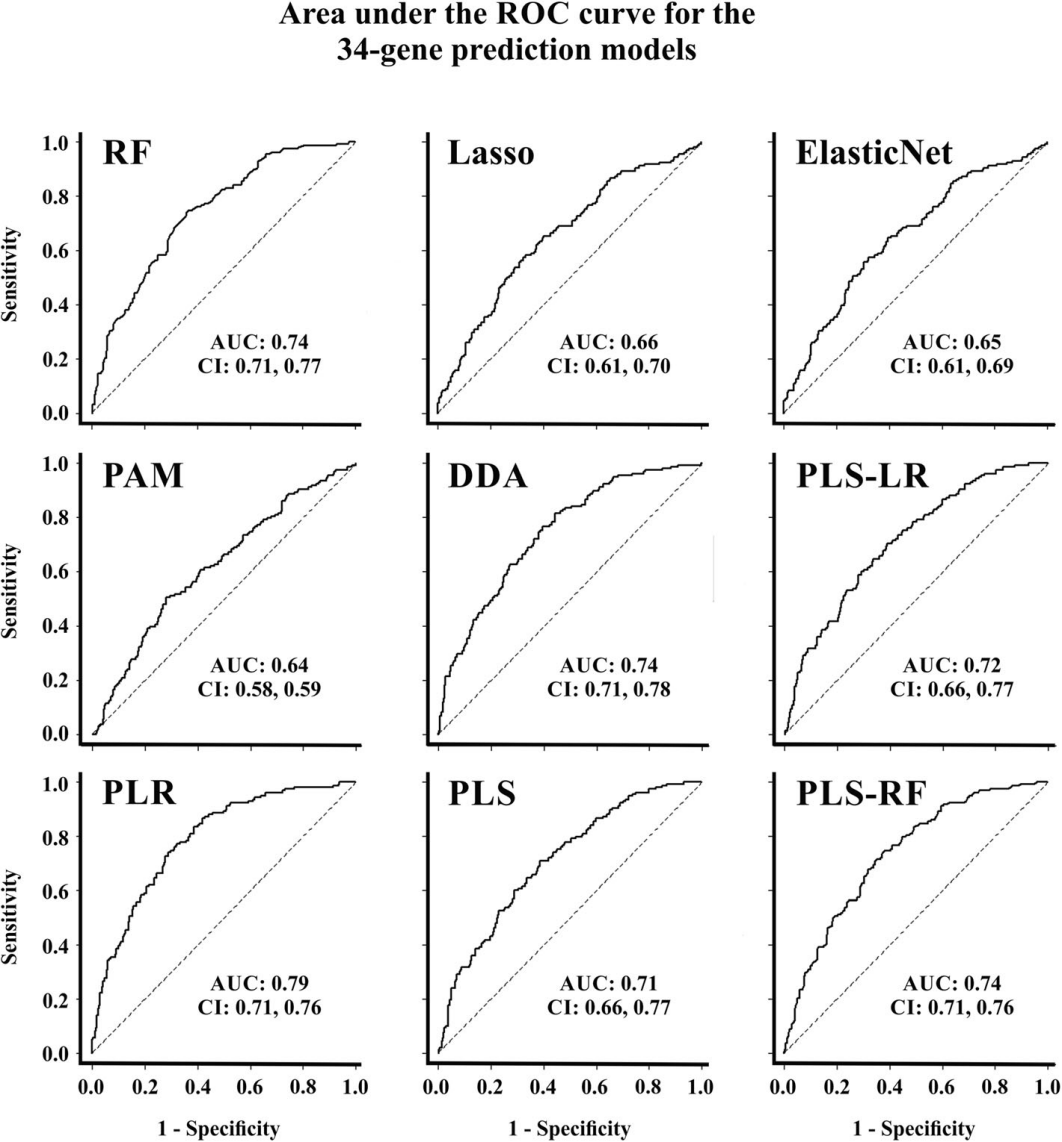
AUC for 34-gene predictions models. AUCs and CIs for the predicting methods using the most relevant genes in the prediction model/classifier: RF: Random forest; Lasso (least absolute shrinkage and selection operator); Elastic Net; PAM: Prediction analysis for microarrays; DDA: Diagonal discriminant analysis; PLS-LR: Partial least squares - Logistic regression; PLR: Penalized logistic regression; PLS: Partial least squares; PLS-RF: Partial least squares - Random forest

The variable selection process identified 105 different genes to be relevant (i.e., at least one ‘hit’) in the prediction model. Of those, only 34 genes had more than 10 hits with all methods of gene selection (at least one hit per method): *RHOT1, MYO7A, ZBTB10, MATK, ST18, RPS23, GCNT1, DROSHA, NUAK1, CCPG1, PDGFD, KLRAP1, MTAP, RNF13, THBS1, MLX, FAP, TIMP3, PRSS1, SLC7A11, PRSS2, OLFML3, RPS20, MCM5, POLE, STEAP4, LRRC8D, C10orf26 (WBP1L), ENTPD5, SYNE1, DPT, COPZ2, TRIO,* and *PDPR*. These were considered to be the most relevant genes for the construction of the model. A new prediction for chemo-response was performed with these 34 genes, termed the optimized 34-gene prediction models, with corrections for batch-effect and clinical variables and using cross-validation (Fig. 3). By selecting only those genes that were most informative in prediction models and removing those that had little or no influence, the performance of the signature in terms of AUC increased across the board by 6–10 % regardless of the specific method.

**Fig. 4:**
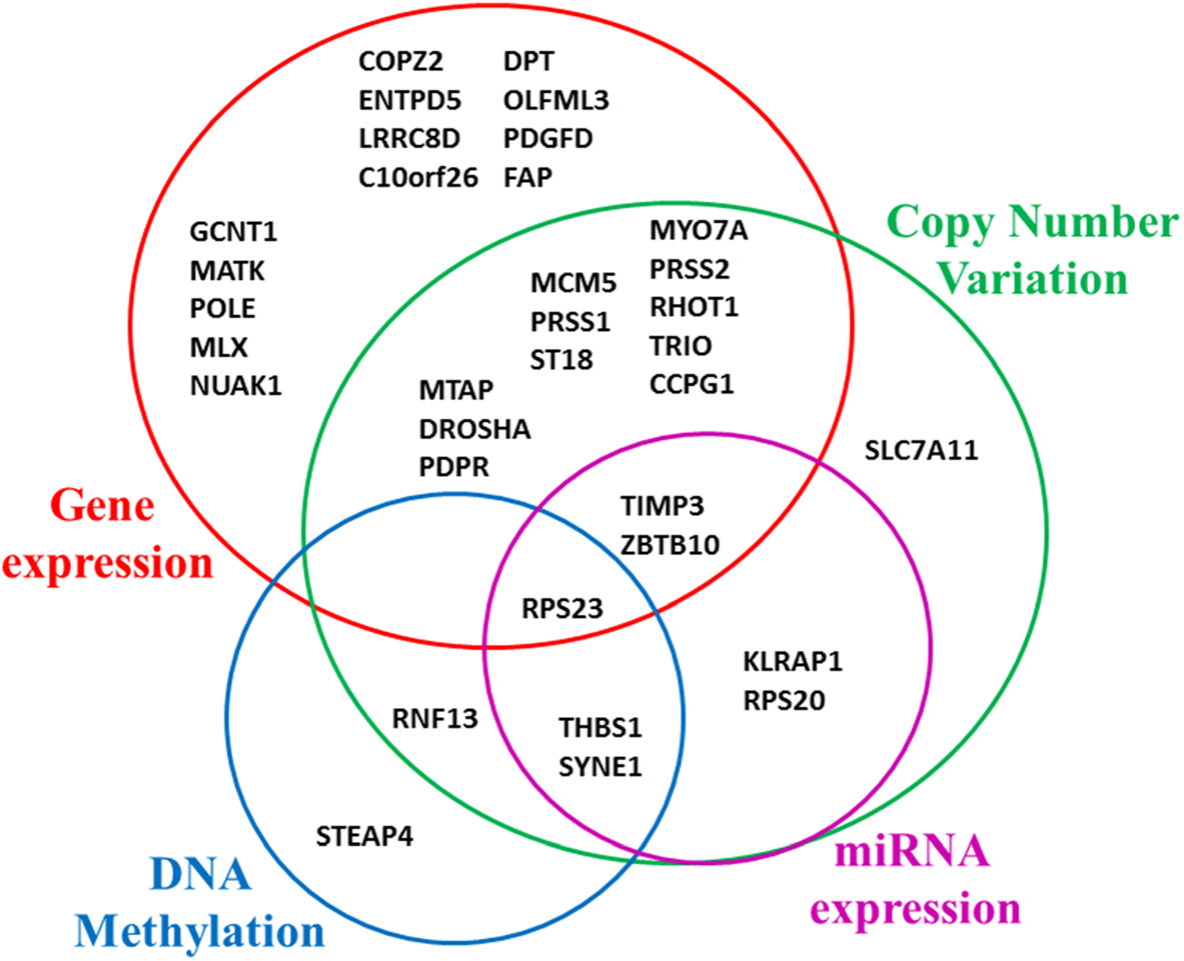
Origin of 34 genes selected in the optimized prediction model. Initially, genes were included in the 422-gene signature because of their differential gene expression (*red*), miRNA expression (*pink*), DNA methylation (*blue*), or copy number variation (*green*) between CR and IR. Some genes had more than one biological difference

**Fig. 5:**
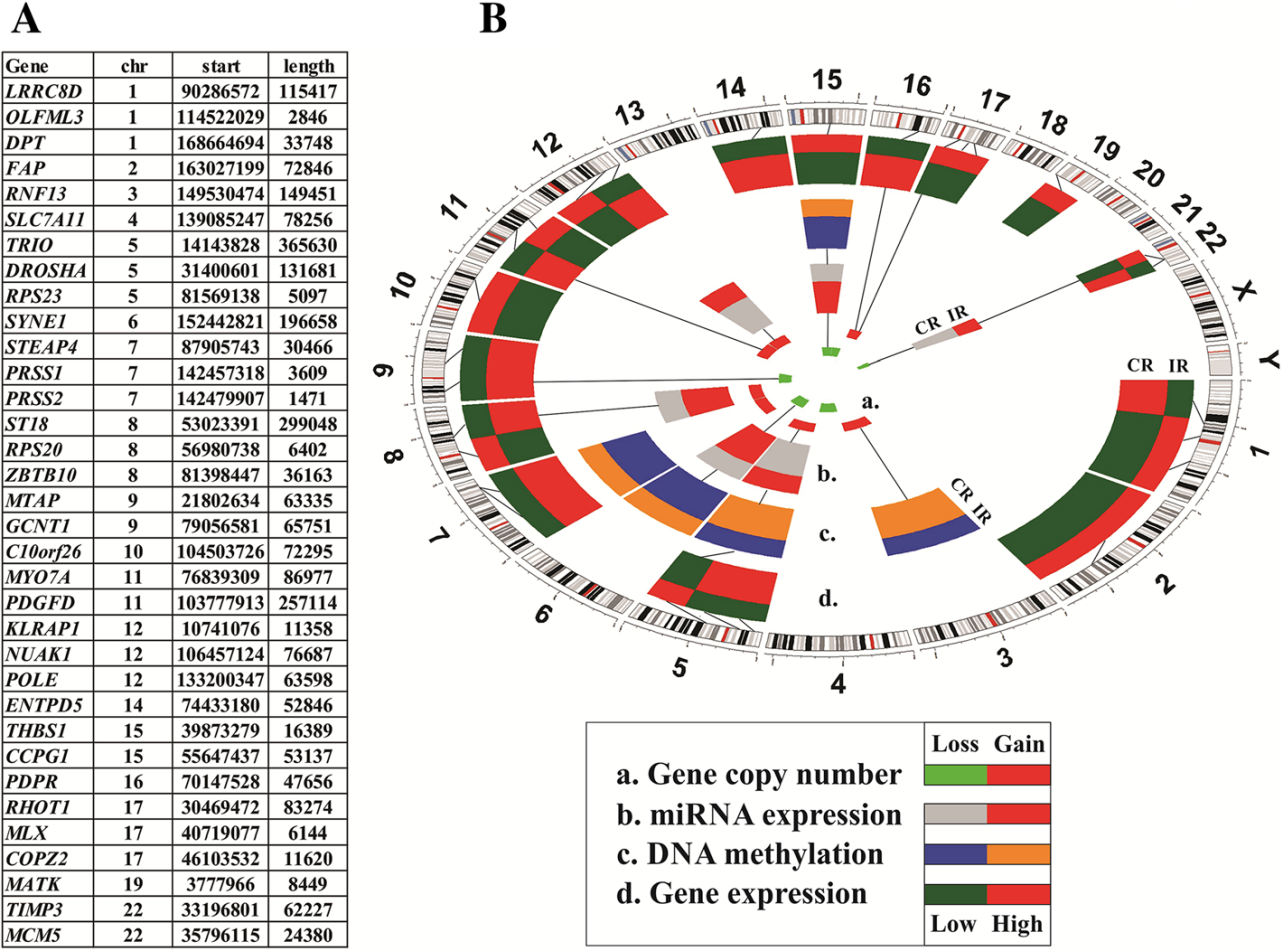
Genomic position of 34 genes selected for the optimized prediction model. **a** The 34 most informative genes from the prediction model and their chromosomal location: chr: number of the chromosome were the gene is located; start: of the gene position; length: of the gene in base-pairs (bp). The human genome version was hg19. **b** Circular layout with matrix depiction of different biological variables. From external to internal: Chromosome bands: circular representation of all chromosomes (centromere is in *red*); *d* Differential gene expression between incomplete and complete responders (CR/IR; *red* is over-expressed, *green* is under-expressed); *c* Differential DNA methylation between CR and IR (CR/IR; *blue* is hypomethylated, *orange* is hyper methylated); *b* Differential miRNA expression between CR and IR (CR/IR; *red* is over-expressed, *grey* is under-expressed); *a* Gene copy number variation between CR and IR (copy gain is *red*, *green* is copy loss). The order of genes in **a** is the same as in **b**. Lines represent correlations between different biological variables (for more details see Table 4)

**Fig. 6:**
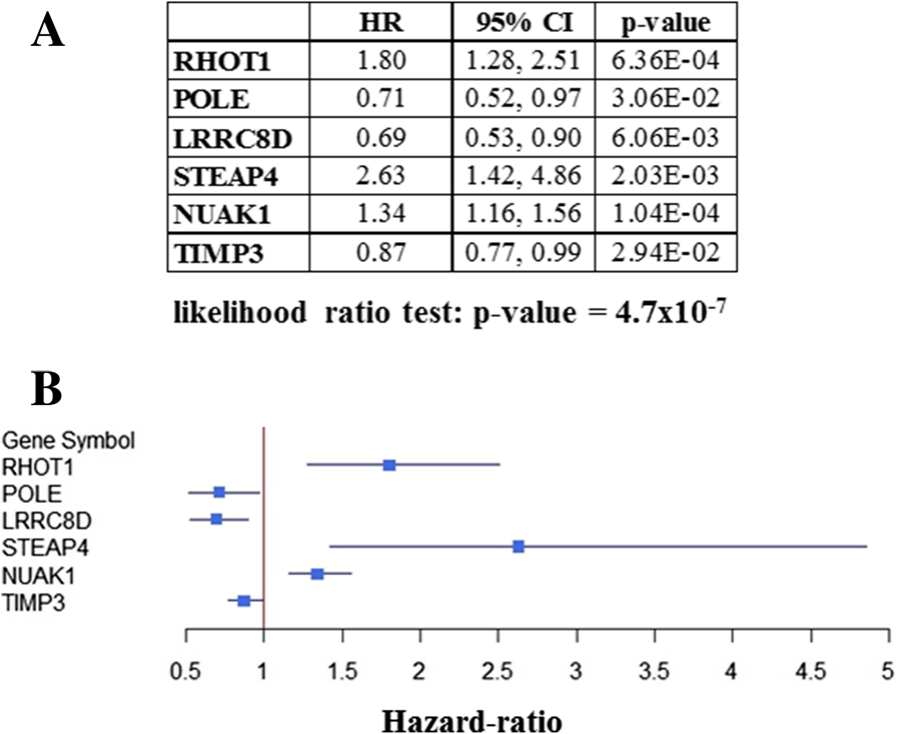
Multivariate survival analysis of the 34 selected genes. **a** Table with hazard-ratio (HR) or risk of death, with 95 % CIs and *p*-values for each of the genes independently associated with survival. **b** Forest plot of independently significant genes for survival. *Blue boxes* represent hazard-ratios (HR), and lines are their CIs. HR = 1 is non-significant. HR < 1 has decreased risk of death; HR > 1 has increased risk of death. Overall *p*-value of the survival model: *p* = 5.7×10^−7^

The 34 selected genes for the optimized prediction model were included in the initial 422-gene signature through different modes: 27 presented differential gene expression between CR and IR, 20 related to copy number variation between CR and IR (14 of them also had differential gene expression), five were correlated with genes with differential DNA methylation between CR and IR, and seven were correlated with miRNAs with different expression between CR and IR (Fig. 4). The relative chromosomal position of the 34 selected genes and the spatial distribution are displayed in Fig. 5 in a circular layout with matrix depiction of their relative expression (Table 4 has detailed information about these 34 genes). Multivariate analysis of survival using Cox proportional hazards regression identified that six of the 34 genes were independently associated with survival (Fig. 6).

**Table 4:** Genomic information and reason for inclusion in the original 422-gene signature for the 34 genes selected in the prediction model

Annotation						Expression	Copy number		DNA Methylation		miRNA expression	
Symbol	Name	Entrez-ID	chr	Start	Length	Fold-change (CR/IR)	Presence	cytoband	Methylated Genes	Methylation Status (CR/IR)	miRNA	CR/IR expression
*LRRC8D*	leucine rich repeat containing 8 family, member D	55144	1	90286572	115417	1.13						
*OLFML3*	olfactomedin-like 3	56944	1	114522029	2846	0.76						
*DPT*	dermatopontin	1805	1	168664694	33748	0.9						
*FAP*	fibroblast activation protein, alpha	2191	2	163027199	72846	0.7						
*RNF13*	ring finger protein 13	11342	3	149530474	149451		Gain	3q22.1-q29	CRKRS	1.19		
*SLC7A11*	solute carrier family 7, (cationic amino acid transporter, y + system) member 11	23657	4	139085247	78256		Loss	4q13.3-q35.2				
*TRIO*	triple functional domain (PTPRF interacting)	7204	5	14143828	365630	1.18	Gain	5p15.33-p13.1				
*DROSHA*	drosha, ribonuclease type III	29102	5	31400601	131681	1.16	Gain	5p15.33-p13.1				
*RPS23*	ribosomal protein S23	6228	5	81569138	5097	0.96	Loss	5q11.2-q21.1	UNQ9217	1.15	miR-22	0.8
*SYNE1*	spectrin repeat containing, nuclear envelope 1	23345	6	152442821	196658		Loss	6q15-q27	LAD1, NFATC2, SLC1A2, STEAP4	0.77	miR-22, miR-200b	1.22
*STEAP4*	STEAP family member 4	79689	7	87905743	30466				SYNE1	0.82		
*PRSS1*	protease, serine, 1 (trypsin 1)	5644	7	142457318	3609	1.32	Gain	7q32.1-q36.3				
*PRSS2*	protease, serine, 2 (trypsin 2)	5645	7	142479907	1471	1.34	Gain	7q32.1-q36.3				
*ST18*	suppression of tumorigenicity 18 (breast carcinoma) (zinc finger protein)	9705	8	53023391	299048	0.97	Gain	8p11.21-q24.3				
*RPS20*	ribosomal protein S20	6224	8	56980738	6402		Gain	8p11.21-q24.3			miR-135b	1.22
*ZBTB10*	zinc finger and BTB domain containing 10	65986	8	81398447	36163	1.2	Gain	8p11.21-q24.3			miR-708	1.2
*MTAP*	methylthioadenosine phosphorylase	4507	9	21802634	63335	1.12	Loss	9p21.3-p21.2				
*GCNT1*	glucosaminyl (N-acetyl) transferase 1, core 2	2650	9	79056581	65751	1.17						
*C10orf26*	WBP1L - chromosome 10 open reading frame 26	54838	10	104503726	72295	0.87	No					
*MYO7A*	myosin VIIA	4647	11	76839309	86977	1.08	Gain	11q13.5-q14.1				
*PDGFD*	platelet derived growth factor D	80310	11	103777913	257114	0.76	No					
*KLRAP1*	killer cell lectin-like receptor subfamily A pseudogene 1	10748	12	10741076	11358		Gain	12p13.33-p11.21			miR-22	0.8
*NUAK1*	NUAK family, SNF1-like kinase, 1	9891	12	106457124	76687	0.75	No					
*POLE*	polymerase (DNA directed), epsilon	5426	12	133200347	63598	1.14	No					
*ENTPD5*	ectonucleoside triphosphate diphosphohydrolase 5	957	14	74433180	52846	1.13	No					
*THBS1*	thrombospondin 1	7057	15	39873279	16389		Loss	15q11.1-q21.1	CORO6, LAD1, NFATC2, SLC1A2, SNAI1	0.74	miR-22, miR-641, miR-200b	1.22
*CCPG1*	cell cycle progression 1	9236	15	55647437	53137	0.84	Loss	15q21.3				
*PDPR*	pyruvate dehydrogenase phosphatase regulatory subunit	55066	16	70147528	47656	1.04	Loss	16q12.2-q24.3				
*RHOT1*	ras homolog gene family, member T1	55288	17	30469472	83274	0.87	Gain	17p13.3-q21.2				
*MLX*	MAX-like protein X	6945	17	40719077	6144	0.88	No					
*COPZ2*	coatomer protein complex, subunit zeta 2	51226	17	46103532	11620	0.78	No					
*MATK*	megakaryocyte-associated tyrosine kinase	4145	19	3777966	8449	0.93	No					
*TIMP3*	TIMP metallopeptidase inhibitor 3	7078	22	33196801	62227	0.7	Loss	22q11.22			miR-22	0.8
*MCM5*	minichromosome maintenance complex component 5	4174	22	35796115	24380	1.19	Loss	22q11.22-q13.33				

In the Expression, Copy number, DNA methylation, and miRNA expression, only those with significant differential values between CR and IR were represented. Some genes had more than one biological difference

Copy number shows the chromosomal region (cytoband) that was significantly correlated with gene expression in the 422-gene signature. DNA Methylation and miRNA expression shows the initial variables that were significantly correlated to gene expression in the 422-gene signature

Pathway enrichment analysis using both GeneGo and clusterProfiler revealed that the 34 genes are particularly relevant to protein absorption and metabolism and DNA repair and replication (Table 5). Also, cytoskeletal remodeling and cell adhesion functions were significantly represented in GeneGo (Table 5), consistent with pathways that are frequently implicated in response to therapy and disease progression.

**Table 5:** Pathway enrichment analysis of the selected 34 genes constituting the simplified prediction model

Cluster Profiler pathway enrichment analysis				
KEGG ID	Description	Adjusted *p*-value	FDR q-value	Gene IDs
hsa03030	DNA replication	2.07E-02	7.28E-03	POLE/MCM5
hsa04974	Protein digestion and absorption	3.09E-02	1.08E-02	PRSS1/PRSS2
hsa03010	Ribosome	3.09E-02	1.08E-02	RPS23/RPS20
hsa00240	Pyrimidine metabolism	3.09E-02	1.08E-02	POLE/ENTPD5
hsa04972	Pancreatic secretion	3.09E-02	1.08E-02	PRSS1/PRSS2
GeneGO pathway enrichment analysis				
#	Description	Adjusted *p*-value	FDR q-value	Gene IDs
1	Cell adhesion_Chemokines and adhesion	8.45E-03	9.91E-02	Thrombospondin 1, TRIO
2	Immune response_IL-12 signaling pathway	3.21E-02	9.91E-02	G6NT
3	Cytoskeleton remodeling_Role of PDGFs in cell migration	3.34E-02	9.91E-02	PDGF-D
4	Triacylglycerol metabolism p.2	3.89E-02	9.91E-02	CEL
5	Development_Thrombospondin-1 signaling	3.89E-02	9.91E-02	Thrombospondin 1
6	Cell cycle_Start of DNA replication in early S phase	4.43E-02	9.91E-02	MCM5
7	Role of Tissue factor in cancer independent of coagulation protease signaling	4.84E-02	9.91E-02	Thrombospondin 1

### Replication of prediction models in independent datasets

After downloading the databases detailed in Table 2, only cases of serous OVCA were selected, and the outcome of interest (chemo-response) was coded to make outcomes comparable with the initial TCGA data set: CR and IR. Initially, we replicated the 422-gene prediction models with cross-validation to avoid over-fitting. Performance of prediction models were measured with AUC (Table 6). AUCs of the prediction models in the independent validation datasets were comparable with the testing set from the TCGA, with CIs overlapping the testing set for almost all methods that contained information from the full 422-gene signature (marked red in Table 6) [32–35].

**Table 6:**
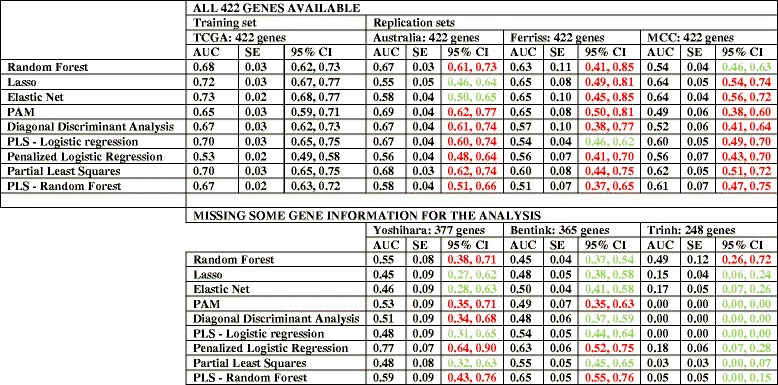
Replication of 422-gene prediction models with different methods

The table presents the results taking in consideration: 1) whether they were part of the training or replication set; 2) the number of genes available for analysis: on top analyses including all 422 genes; on the bottom analyses where all genes were not available (and the number included)

CI of replication datasets with WIDE overlap with TCGA testing set CI: in red. CI of replication datasets with NO overlap with TCGA testing set CI: in green. AUC: area under the ROC curve. CI: confidence intervals. SE: Standard Error. PAM: Prediction analysis for microarrays; PLS: Partial least squares; Lasso: least absolute shrinkage and selection operator

Unfortunately, all genes were not available in all platforms from the independent datasets, and three independent datasets had incomplete gene information [36–38]. The performance of these incomplete datasets was inferior to the TCGA classifier, with AUCs closer to 50 % (marked green in Table 6; see also Additional file 1: Table S1 for more details about classifier performance in relation to sensitivity, specificity, and standard errors in all available databases). Thus, the incomplete information in some of the replication sets provided insight into how well prediction models performed when some of the genes were not available.

### Validation of prediction models in independent datasets

Validation of 422-gene prediction models were performed using TCGA data as the training set and independent datasets that contained complete information for all 422-genes as the testing sets. AUC and their CIs were used to validate the performance of the TCGA classifier in the three independent datasets with all gene expression information (Table 7). CIs of the AUC in the three different validation sets overlapped with the majority of the CIs of TCGA testing set (marked red in Table 7), validating the use of the 422-gene signature to predict chemo-response.

**Table 7:**
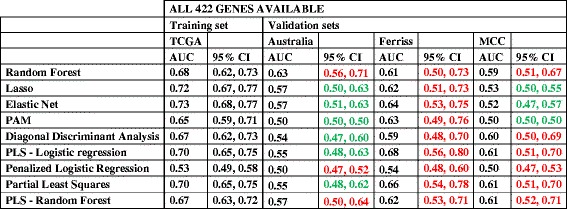
Validation of 422-gene prediction models in independent databases

The table presents the results taking into consideration whether they were part of the training or validation set

CI of validation datasets WITH overlap with TCGA testing set CI: in red. CI of validation datasets with NO overlap with TCGA testing set CI: in green. AUC: area under the ROC curve. CI: confidence intervals. SE: Standard Error. PAM: Prediction analysis for microarrays; PLS: Partial least squares; Lasso: least absolute shrinkage and selection operator

Next, we validated TCGA 34-gene prediction models in the same three independent datasets with all gene information, that also included all the most informative 34 genes selected from the training set. Analysis of AUCs and CIs demonstrated that the optimized model performed very well in these validations sets, with AUC values ranging from 56 to 73 % and overlap of the majority of the CIs with the testing test (in red in Table 8). However, the CIs were wider in the validation set than in the testing set. For details about prediction models performance in relation to sensitivity, specificity, and standard deviation in all available databases see Additional file 2: Table S2.

**Table 8:**
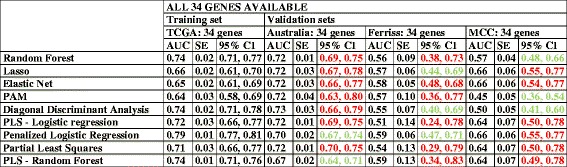
Validation of optimized 34-gene prediction models

The table presents the results taking into consideration whether they were part of the training or validation set

CI of validation datasets WITH overlap with TCGA testing set CI: in red. CI of validation datasets with NO overlap with TCGA testing set CI: in green. AUC: area under the ROC curve. CI: confidence intervals. SE: Standard Error. PLS: Partial least squares

### Clinical characteristics associated with misclassification in prediction models

Additional file 1: Tables S1 and Additional file 2: Table S2 list the misclassification rate for each of the prediction models in all analyzed datasets. To assess whether the prediction models could be further improved, we excluded those patients at high risk of misclassification and the clinical characteristics that are associated with this misclassification. First, we identified those samples that missed the true classification (either CR or IR) more than 20 % of the time on average: 158 of 450 patients in TCGA dataset. Next, we examined which baseline clinic characteristics (e.g., grade, stage, optimal debulking) were independently associated with misclassification using a multivariable regression analysis. The only independent variable associated with misclassification was ‘optimal treatment’ (*p* < 0.001), defined as treatment with optimal cytoreductive surgery and 6 cycles of platinum-based chemotherapy. Unfortunately, TCGA was not designed to study chemo-response, and other variables that may have affected the delivery of an optimal treatment were not collected and thus could not be included in the prediction models.

## Discussion

Initial response to chemotherapy remains one of the most significant prognostic markers for serous OVCA patients. Identifying patients at high risk of chemoresistance early in the course of treatment has the potential to significantly alter clinical management, such as performing in depth tumor sequencing, more frequent disease monitoring or exploration of additional therapeutic options. Previous studies have focused in prediction models that classify ovarian cancer patients in prognostic groups [9, 10, 17]. While this is very valuable to clinicians and patients to assess the severity of the disease, prognosticators have limited immediate clinical application and they may not translate into effective therapeutic strategies. The design of our study was aimed to have immediate effect on treatment decisions. Using data available in TCGA for serous OVCA, we previously identified a 422-gene signature that is associated with chemo-response [18]. However, in order to develop this signature into a clinically useful test, it was paramount to establish its predictive performance. Herein we report the performance and validation of the 422-gene and optimized 34-gene models using TCGA and six other datasets. Specifically, 422-gene prediction models in the testing dataset (TCGA) presented AUCs around 70 %, and 34-gene predictive models performed slightly better, with AUCs above 70 %. This represents a significant improvement over current clinical estimations of complete chemo-response, which are between 40 and 60 % [3], setting the stage for the development of the 34-gene predictive model as a test to identify patients with serous OVCA at high risk for treatment failure.

The bulk of OVCA research is directed at understanding the biology of the disease [5, 6] and defining subsequent treatment for those who do not respond or recur after initial chemotherapy [16]. By comparison, we have a very poor understanding of which patients are at risk of failing initial treatment, due in large part to a lack of validated biomarkers or molecular signatures that can effectively predict chemo-response [7, 8]. Previous attempts have been limited by the number of patients included in the study, analysis of a mixture of histological types and stages, and lack of validation in independent datasets [9, 10, 16]. Some of these prediction models included serum levels of CA125, a tumor-associated glycoprotein of unknown function that is used clinically to assess disease burden, though it has limited value in predicting chemo-response [9, 10, 39]. In the present study, we have created prediction models of chemo-response from a 422-gene signature comprising only serous cases, the most common type of OVCA. These prediction models not only had AUCs over 70 %, but were replicated and validated in the largest collection of independent serous OVCA databases available to date.

## Conclusions

One of the major strengths of this study is the replication and validation of prediction models in large independent datasets containing gene expression specific to serous OVCA and information about response to chemotherapy. However, it is important to recognize the limitations inherent in the retrospective design of the databases used to create and validate the prediction models, especially since these databases were not originally intended to study chemo-response as a primary outcome variable. Although this clinical parameter was recorded in all databases, it might contain biases. To minimize this, we used a strict definition of chemo-response to segregate patients into CR and IR. Regardless of these limitations, we demonstrate replication and validation of both the 422-gene and the 34-gene models across multiple databases, with overlapping CIs. Of particular significance is the validation of the optimized 34-gene models given the variability in gene expression analysis among different datasets. This highlights the utility of this gene signature as a *bona fide* indicator of chemo-response.

Identification of characteristics independently associated with misclassification could be very helpful in improving prediction models. For example, if we identify that patients with BMI > 50 are more likely to be misclassified, we might exclude those patients from the prediction process to enhance the classification of the other patients. Also, based on the observations made with patients with optimal treatment, fine-tuning of the prediction models may be possible, and perhaps necessary. Researchers in cardiovascular disease have been at the forefront of developing risk prediction equations utilizing clinical risk factors for the prediction of cardiovascular events, as in the Framingham Study [40]. Recently, new biomarkers and nonclinical measures have been studied to improve these risk prediction tools in the population [41]. Adding new biomarkers to the classic clinical risk factors may improve the model and offer better prediction of the 15–20 % of patients with cardiovascular events that have no clinical risks before the episode [41]. In OVCA, we found that there was one variable associated with misclassification, ‘optimal treatment’. Variables associated with the adequate delivery of optimal treatment in OVCA (surgery and chemotherapy) have the potential to improve prediction models significantly [42]. Quantification and different metrics for optimal treatment delivery could be determined in a prospective set of patients with comprehensive clinical data collection in order to better understand which factors influence treatment outcome.

A caveat to our study is that datasets with incomplete data performed poorly, which was accentuated in models with 34 genes. For example, in the Yoshihara dataset [37], we only lost expression information for one of the seven more influential genes in the model. However, this gene (*MATK*) was ranked second in importance, so the model underperformed. The Bentink database [38] lost fewer genes that Yoshihara (12 versus 14) but two of them were seven of the most influential genes, and the performance was inferior to the Yoshihara database. Trihn databse [36] lost four of the seven most influential genes, including the first two (*RHOT1* and *MATK*), with serious consequences for its performance. Although this loss of performance could be seen as a problem for the construction of our prediction models, it also underscores that the importance of the genes included in the model is proportional to its performance, thereby supporting the robust performance of the 34-gene prediction models. Therefore, we propose that these prediction models are very promising and are robust across different databases and classifiers. Nevertheless, additional validation analyses are necessary to test their utility as a clinical test for response to chemotherapy in OVCA.

As to the pathways associated with the 422-gene signature, the majority of the 34 selected genes included in the optimized prediction model are drawn from cellular functions previously associated with response to chemotherapy [8, 18]. Specifically, the pathway enrichment analysis identified pathways involved in DNA damage repair, replication, protein metabolism, cell cycle and apoptosis, as well as cytoskeletal remodeling and cell adhesion functions, all of which have been associated with cancer transformation and proliferation [43]. Several genes in our optimized model have potential implications relative to the etiology or treatment of serous OVCA. For example, mutations in DNA polymerase epsilon (*POLE*) have been reported in endometrial and colon cancers with elevated numbers of somatic mutations [44, 45], which appears to correlate with a robust intratumoral T-cell response and better prognosis [46]. To date, however, there have been no reports of *POLE* differential gene expression and its association with prognosis in serous OVCA. Decreased expression of *GCNT1* [47], *DROSHA* [48] and increased expression of *TIMP3* [49] have been associated with decreased PFS and OS in independent analyses of TCGA. Of note, we found that POLE, LRRC3D, and TIMP3 mutations were all associated with improved survival.

Other genes, like *FAP,* have been associated with clinical resistance to chemotherapy in patients with serous OVCA, which carries worse prognosis and survival [50]. Although there is no direct evidence of clinical association with progression of disease in serous ovarian cancer, there are in vitro studies that implicate *NUAK1* (*ARK5*) with invasion and progression in ovarian cancer cell lines [51]. *ARK5* expression has been found to be elevated in serous OVCA, and its expression has been associated with invasion and metastasis in cancer in general [51]. Supporting the role of NUAK1/ARK5 in the aggressive cancer phenotype, this gene was independently associated with decreased survival (HR = 1.34, 95 % CI 1.16–1.56).

We also identified two genes, thrombospondin I (*THBS1*) and *PDGFD*, which participate in a wide range of signaling pathways that regulate cellular processes involved in cancer genesis and progression [52]. Thrombospondin I is a secreted protein that associates with the extracellular matrix and possesses a variety of biologic functions, including having potent antiangiogenic activity. In OVCA, decreased production of thrombospondin I results in increased expression of vascular endothelial growth factor (VEGF) and other angiogenic factors such as P1GF, FGF-2 and PDGFB [53]. *PDGFD,* also a member of the platelet-derived growth factor (PDGF) family, participates in cytoskeletal remodeling and, through its role in cell migration, contributes to vascular integrity and function mediated by perycite coverage of vessels. This action could be disrupted in tumors [43]. Also, platelet levels during treatment and recruitment to tumors have been associated with chemo-response [54]. Taken together, these data may provide a molecular basis for the efficacy of bevacizumab, a humanized antibody against *VEGF*, which has been shown to improve progression-free survival and even overall survival in a subset of serous OVCA patients [55, 56], or the use of multitarget tyrosine kinase inhibitors to simultaneously block *VEGFR*, *PDGFR* and *FGFR* pathways [57].

Up to a third of the patients undergoing treatment for advanced serous OVCA do not respond to treatment and have a very poor prognosis. Indeed, more than 40 % of the patients in this poor prognosis group have had optimal therapy, with optimal cytoreductive surgery and at least six cycles of platinum-based chemotherapy, indicating that lack of response is not due to incomplete clinical treatment. Hence, for many such patients, the inherit characteristics of the tumor appear to play a major role in lack of responsiveness. We have created 34-gene prediction models/classifiers that consistently predict these patients approximately 80 % of the time. This prediction model is specific for patients diagnosed with serous OVCA. Our ultimate goal is to create a diagnostic tool that will predict each patient’s response to chemotherapy. This is important, because, based on the potential molecular targets identified in the 34-gene prediction models, we could design clinical trials that compare standard therapy versus standard therapy in combination with molecular agents targeting resistance pathways for the women who are unlikely to respond to the usual upfront regimens.

## Additional files

Additional file 1: Table S1. AUCs, CI, sensitivity, specificity and misclassification rate of testing and validation of 422-gene prediction models. (DOCX 32 kb)
Additional file 2: Table S2. AUCs, CI, sensitivity, specificity and misclassification rate of testing and validation for 34-gene prediction models. (DOCX 25 kb)

## Abbreviations

AUC: Area under the curves
BRB: Biometric research branch
CI: Confidence interval
CMA: Classification for MicroArrays
CR: Complete responders
EMBL-EBI: European Bioinformatics Institute, part of the European Molecular Biology Laboratory
FDR: False discovery rate
FIGO: International Federation of Obstetrics and Gynecology
IR: Incomplete responders
GEO: Gene expression omnibus
HR: Hazard-ratio
KEGG database: Kyoto encyclopedia of genes and genomes
Lasso: Least absolute shrinkage and selection operator
miRNA: micro RNA
NCI: National Cancer Institute
NHGRI: National Human Genome Research Institute
OVCA: Serous ovarian cancer
PAM: Prediction analysis for microarrays
PLS: Partial least squares
RFE: Recursive feature elimination
ROC: Receiver operating characteristic
SVM: Support vector machines
TCGA: The Cancer Genome Atlas
