# Supplementary material for: Prediction of chemo-response in serous ovarian cancer
Source: Mol Cancer. 2016 Oct 19;15:66. doi: 10.1186/s12943-016-0548-9 (PMC5070116; doi:10.1186/s12943-016-0548-9)
Supplement: Additional file 1: Table S1. — AUCs, CI, sensitivity, specificity and misclassification rate of testing and validation of 422-gene prediction models. (DOCX 32 kb) [file 12943_2016_548_MOESM1_ESM.docx]

**Supplementary Table S1:** **AUCs, CI, sensitivity, specificity and misclassification rate of testing and validation of 422-gene prediction models.**

| **TCGA 422 genes** | | | | | | | |
| --- | --- | --- | --- | --- | --- | --- | --- |
|  | **misclassification** | **sensitivity** | **specificity** | **AUC** | **SE** | **High**  **95% CI** | **Low**  **95% CI** |
| **Random Forest** | 0.35 | 0.13 | 0.93 | 0.68 | 0.03 | 0.62 | 0.73 |
| **Lasso** | 0.31 | 0.48 | 0.80 | 0.72 | 0.03 | 0.67 | 0.77 |
| **Elastic Net** | 0.30 | 0.45 | 0.82 | 0.73 | 0.02 | 0.68 | 0.77 |
| **PAM** | 0.34 | 0.27 | 0.87 | 0.65 | 0.03 | 0.59 | 0.71 |
| **Diagonal Discriminant Analysis** | 0.36 | 0.58 | 0.67 | 0.67 | 0.03 | 0.62 | 0.73 |
| **PLS - Logistic regression** | 0.34 | 0.41 | 0.80 | 0.70 | 0.03 | 0.65 | 0.75 |
| **Penalized Logistic Regression** | 0.48 | 0.49 | 0.53 | 0.53 | 0.02 | 0.49 | 0.58 |
| **Partial Least Squares** | 0.34 | 0.41 | 0.80 | 0.70 | 0.03 | 0.65 | 0.75 |
| **PLS - Random Forest** | 0.36 | 0.41 | 0.77 | 0.67 | 0.02 | 0.63 | 0.72 |
|  | | | | | | | |
| **Australia 422 genes** | | | | | | | |
|  | **misclassification** | **sensitivity** | **specificity** | **AUC** | **SE** | **High 95% CI** | **Low 95% CI** |
| **Random Forest** | 0.22 | 0.05 | 1.00 | 0.67 | 0.03 | 0.61 | 0.73 |
| **Lasso** | 0.30 | 0.15 | 0.86 | 0.55 | 0.05 | 0.46 | 0.64 |
| **Elastic Net** | 0.28 | 0.14 | 0.90 | 0.58 | 0.04 | 0.50 | 0.65 |
| **PAM** | 0.29 | 0.52 | 0.76 | 0.69 | 0.04 | 0.62 | 0.77 |
| **Diagonal Discriminant Analysis** | 0.33 | 0.68 | 0.68 | 0.67 | 0.04 | 0.61 | 0.74 |
| **PLS - Logistic regression** | 0.26 | 0.26 | 0.88 | 0.67 | 0.04 | 0.60 | 0.74 |
| **Penalized Logistic Regression** | 0.35 | 0.31 | 0.76 | 0.56 | 0.04 | 0.48 | 0.64 |
| **Partial Least Squares** | 0.28 | 0.26 | 0.87 | 0.68 | 0.03 | 0.62 | 0.74 |
| **PLS - Random Forest** | 0.25 | 0.25 | 0.90 | 0.58 | 0.04 | 0.51 | 0.66 |
|  | | | | | | | |
| **Ferriss 422 genes** | | | | | | | |
|  | **misclassification** | **sensitivity** | **specificity** | **AUC** | **SE** | **High 95% CI** | **Low 95% CI** |
| **Random Forest** | 0.44 | 0.24 | 0.83 | 0.63 | 0.11 | 0.41 | 0.85 |
| **Lasso** | 0.44 | 0.32 | 0.77 | 0.65 | 0.08 | 0.49 | 0.81 |
| **Elastic Net** | 0.45 | 0.28 | 0.77 | 0.65 | 0.10 | 0.45 | 0.85 |
| **PAM** | 0.46 | 0.56 | 0.61 | 0.65 | 0.08 | 0.50 | 0.81 |
| **Diagonal Discriminant Analysis** | 0.40 | 0.68 | 0.57 | 0.57 | 0.10 | 0.38 | 0.77 |
| **PLS - Logistic regression** | 0.49 | 0.45 | 0.61 | 0.54 | 0.04 | 0.46 | 0.62 |
| **Penalized Logistic Regression** | 0.40 | 0.43 | 0.72 | 0.56 | 0.07 | 0.41 | 0.70 |
| **Partial Least Squares** | 0.42 | 0.45 | 0.73 | 0.60 | 0.08 | 0.44 | 0.75 |
| PLS - Random Forest | 0.50 | 0.38 | 0.62 | 0.51 | 0.07 | 0.37 | 0.65 |
|  | | | | | | | |
| **MCC 422 genes** | | | | | | | |
|  | **misclassification** | **sensitivity** | **specificity** | **AUC** | **SE** | **High 95% CI** | **Low 95% CI** |
| **Random Forest** | 0.30 | 0.02 | 0.98 | 0.54 | 0.04 | 0.46 | 0.63 |
| **Lasso** | 0.33 | 0.26 | 0.85 | 0.64 | 0.05 | 0.54 | 0.74 |
| **Elastic Net** | 0.32 | 0.15 | 0.89 | 0.64 | 0.04 | 0.56 | 0.72 |
| **PAM** | 0.30 | 0.00 | 0.99 | 0.49 | 0.06 | 0.38 | 0.60 |
| **Diagonal Discriminant Analysis** | 0.41 | 0.38 | 0.69 | 0.52 | 0.06 | 0.41 | 0.64 |
| **PLS - Logistic regression** | 0.33 | 0.26 | 0.83 | 0.60 | 0.05 | 0.49 | 0.70 |
| **Penalized Logistic Regression** | 0.37 | 0.30 | 0.77 | 0.56 | 0.07 | 0.43 | 0.70 |
| **Partial Least Squares** | 0.33 | 0.24 | 0.84 | 0.62 | 0.05 | 0.51 | 0.72 |
| **PLS - Random Forest** | 0.33 | 0.30 | 0.82 | 0.61 | 0.07 | 0.47 | 0.75 |
|  | | | | | | | |
| **Yoshihara 377 genes** | | | | | | | |
|  | **misclassification** | **sensitivity** | **specificity** | **AUC** | **SE** | **High 95% CI** | **Low 95% CI** |
| **Random Forest** | 0.15 | 0.00 | 1.00 | 0.55 | 0.08 | 0.38 | 0.71 |
| **Lasso** | 0.17 | 0.03 | 0.96 | 0.45 | 0.09 | 0.27 | 0.62 |
| **Elastic Net** | 0.16 | 0.00 | 0.99 | 0.46 | 0.09 | 0.28 | 0.63 |
| **PAM** | 0.15 | 0.00 | 1.00 | 0.53 | 0.09 | 0.35 | 0.71 |
| **Diagonal Discriminant Analysis** | 0.31 | 0.28 | 0.77 | 0.51 | 0.09 | 0.34 | 0.68 |
| **PLS - Logistic regression** | 0.18 | 0.18 | 0.93 | 0.48 | 0.09 | 0.31 | 0.65 |
| **Penalized Logistic Regression** | 0.16 | 0.32 | 0.93 | 0.77 | 0.07 | 0.64 | 0.90 |
| **Partial Least Squares** | 0.18 | 0.08 | 0.94 | 0.48 | 0.08 | 0.32 | 0.63 |
| **PLS - Random Forest** | 0.16 | 0.18 | 0.95 | 0.59 | 0.09 | 0.43 | 0.76 |
|  | | | | | | | |
| **Bentink 365 genes** | | | | | | | |
|  | **misclassification** | **sensitivity** | **specificity** | **AUC** | **SE** | **High 95% CI** | **Low 95% CI** |
| **Random Forest** | 0.46 | 0.00 | 0.90 | 0.45 | 0.04 | 0.37 | 0.54 |
| **Lasso** | 0.40 | 0.36 | 0.73 | 0.48 | 0.05 | 0.38 | 0.58 |
| **Elastic Net** | 0.44 | 0.30 | 0.73 | 0.50 | 0.04 | 0.41 | 0.58 |
| **PAM** | 0.39 | 0.00 | 1.00 | 0.49 | 0.07 | 0.35 | 0.63 |
| **Diagonal Discriminant Analysis** | 0.51 | 0.34 | 0.59 | 0.48 | 0.06 | 0.37 | 0.59 |
| **PLS - Logistic regression** | 0.44 | 0.44 | 0.61 | 0.54 | 0.05 | 0.44 | 0.64 |
| **Penalized Logistic Regression** | 0.39 | 0.50 | 0.68 | 0.63 | 0.06 | 0.52 | 0.75 |
| **Partial Least Squares** | 0.44 | 0.44 | 0.59 | 0.55 | 0.05 | 0.45 | 0.65 |
| **PLS - Random Forest** | 0.37 | 0.40 | 0.77 | 0.65 | 0.05 | 0.55 | 0.76 |
|  | | | | | | | |
| **Trinh 248 genes** | | | | | | | |
|  | **misclassification** | **sensitivity** | **specificity** | **AUC** | **SE** | **High 95% CI** | **Low 95% CI** |
| **Random Forest** | 0.40 | 0.91 | 0.18 | 0.49 | 0.12 | 0.26 | 0.72 |
| **Lasso** | 0.78 | 0.30 | 0.13 | 0.15 | 0.04 | 0.06 | 0.24 |
| **Elastic Net** | 0.80 | 0.33 | 0.05 | 0.17 | 0.05 | 0.07 | 0.26 |
| **PAM** | 0.86 | 0.23 | 0.00 | 0.00 | 0.00 | 0.00 | 0.00 |
| **Diagonal Discriminant Analysis** | 0.92 | 0.08 | 0.03 | 0.00 | 0.00 | 0.00 | 0.00 |
| **PLS - Logistic regression** | 0.82 | 0.28 | 0.00 | 0.00 | 0.00 | 0.00 | 0.00 |
| **Penalized Logistic Regression** | 0.76 | 0.27 | 0.18 | 0.18 | 0.06 | 0.07 | 0.28 |
| **Partial Least Squares** | 0.82 | 0.28 | 0.00 | 0.03 | 0.03 | 0.00 | 0.07 |
| **PLS - Random Forest** | 0.78 | 0.37 | 0.00 | 0.05 | 0.05 | 0.00 | 0.15 |

PAM: Prediction analysis for microarrays; PLS: Partial least squares; Lasso: least absolute shrinkage and selection operator.
